# Prediction of solubility of hydrogen in chemicals using QSPR-based machine learning approach: a comparative study

**DOI:** 10.1007/s10822-026-00899-y

**Published:** 2026-07-29

**Authors:** Ali Ebrahimpoor Gorji, Ville Alopaeus

**Affiliations:** School of Chemical Engineering, Department of Chemical and Metallurgical Engineering, Aalto University, Research Group of Chemical Engineering, P.O. Box 16100, 00076 Aalto, Finland

**Keywords:** Solubility of H_2_ in chemicals, Machine learning (ML), Data-driven QSPR method, Prediction, Temperature and pressure effects

## Abstract

The ‘Quantitative Structure–Property Relationship’ (QSPR) method has been used for the prediction of solubility of hydrogen (x) in different chemicals. The dataset consists of 3761 datapoints including 100 unique chemicals at the wide ranges of T and P. An MLR-model, the simplest form of machine learning algorithm, was constructed using the selected descriptors to predict x in various chemicals. For the first time, a comprehensive and predictive MLR-based QSPR model has been developed for this target. The dataset was divided into a training set including 2570 datapoints and to a test set including 1191 datapoints. The advantages of this approach are thoroughly discussed and compared with other available models which were developed with other ML algorithms. Unlike previous models, internal validation was performed on the MLR-QSPR model. According to the results of statistical parameters (R^2^ = 0.96 and Q^2^_LOO-CV_ = 0.96), the predictive capability of the MLR-QSPR model was acceptable for training set.

**Supplementary Information:**

The online version contains supplementary material available at 10.1007/s10822-026-00899-y.

## Introduction

There is an increasing effort to address the challenges caused by the large-scale emission of CO_2_ into the atmosphere. For instance, the search for renewable energy sources like solar irradiation [1] and hydrogen (H_2_) [2] is highly prioritized to help mitigate global warming. For this regard, H_2_ has recently attracted considerable attention from researchers [3–6] compared to other energy sources.

In one hand, a thorough understanding of the thermodynamic properties of H_2_, particularly the solubility of H_2_ in various chemicals as one of important properties, is essential for the effective design of storage systems [7]. On the other hand, precise knowledge of phase equilibria within geological reservoirs is essential for studying mobility, H_2_ reactivity, and as well as for the control, monitoring, and optimization of storage systems [7]. Consequently, an accurate understanding of solubility of H_2_ in wide ranges of chemicals is critical for the development of hydroprocessing techniques [8] and various industrial applications [5].

According to 62 literature studies [8–69], three primary factors influencing effectiveness, temperature (T), pressure (P), and the type of chemicals, have been experimentally studied on the solubility of H_2_ in chemicals. In summary, the solubility of H_2_ in 101 chemicals of 11 distinct types of chemicals such as aliphatic hydrocarbon, alcohol, aromatic hydrocarbon has been studied. Operational conditions (i.e., T and P) in these studies could reach up to 650 K, and 6000 bar, respectively. In one of most recent studies [70], a comprehensive machine learning (ML) framework was developed to predict solubility of H_2_ in 96 different chemicals, utilizing an extensive dataset of 3726 experimental datapoints. Various algorithms of ML including decision tree (DT), multilayer perceptron (MLP), random forest (RF), and extremely randomized trees (ET), were employed. From the statistical metrics viewpoint, the ET algorithm demonstrated superior performance in comparison with other algorithms, achieving high predictive accuracy, thereby validating the robustness and generalizability of the approach. These findings contribute to a deeper understanding of solubility behavior of H_2_ across diverse chemical types and represent a substantial advancement in data-driven modelling for industrial and renewable applications. However, the previous study [70] exhibited several limitations that require careful attention. These include the selection and nature of input variables, the availability and practical usability of these inputs, appropriate screening and classification of chemicals based on input relevance, and the assessment of intercorrelations among input variables. Additionally, there were noticeable discrepancies between the reported statistical parameters for the training and test sets, highlighting the need for a more rigorous comparison and validation of model performance across both sets. Furthermore, the development and evaluation of a multilinear regression (MLR) model representing the simplest ML algorithm were not adequately addressed. Therefore, the present study aims to systematically address these gaps by refining input selection, improving feature analysis, rigorously validating models on both training and test sets, and incorporating a robust MLR approach for comparative purposes. Apart from the above study [70], previous attempts have utilized equations of state (EOSs) to predict the solubility of H_2_ in hydrocarbons. However, the accuracy of these EOSs has been limited, particularly under conditions involving high pressure and/or high temperature [5, 6]. As a result, various ML algorithms as the alternative and complement methods have been employed to enhance predictive performance. Several ML techniques, including DT, RF, multiple linear regression (MLR), multiple nonlinear regression (MNLR), k-nearest neighbors (kNN), gradient boosting machine (GBM), support vector machine (SVM), and artificial neural networks (ANN), can been explored for this purpose [71]. A thorough review of the literature on these modeling approaches for predicting solubility of H_2_ across different chemicals such as aliphatic and aromatic hydrocarbons and alcohols has been conducted, with a summary of key studies presented in Table 1. Notably, most of above ML algorithms, with the exception of MLR, have been more actively studied in the past four years. Our former study is the only study including MLR model in the literature which was focused on 32 hydrocarbons (see Table 1). For comparative purposes, relevant statistical parameters are provided for each ML method wherever available.

**Table 1:** Numbers of datapoints and studied chemicals at each former dataset, and different used ML algorithms for the prediction of solubility of H_2_ in the different chemicals with their independent inputs and their values of statistical parameters

Research group (year)	Number of datapoints	Studied chemicalsin the dataset	Independent variables (inputs)	Applied ML algorithms	Statistical parameters				Ref
					R^*2*^	RMSE	MSE	AARD%	
Gorji and Alopaeus (2024)	1751	*32 Hydrocarbons*: Methane, Ethane, propane, butane, pentane, hexane, heptane, octane, decane, dodecane, hexadecane, eicosane, octacosane, hexatriacontane, hexatetracontane, 2,2,4 trimethylpentane, ethene, 1-octene, benzene, toluene, ethylbenzene, m-xylene, cumene, 1,2,4 trimethylbenzene, diphenylmethane, cyclohexane, methylcyclohexane, naphthalene, 1,2,3,4 tetrahydronaphthalene, phenanthrene, pyrene, squalane	T, P, and four PaDEL descriptors: ATSC0m, MATS1e, minssCH2, ETA_Beta_s	Multilinear Regression (MLR)	0.9400^a^	0.0188^a^	–	–	[72]
			T, P, and six sigma profile descriptors	Multilinear Regression (MLR)	0.9000^a^	0.0224^a^	–	–	
Foroughizadeh et.al (2024)	3726	*96 chemicals*: including n-alkanes, aliphatic and aromatic hydrocarbons, alcohols, aldehydes, diols, esters, ethers, ketones, and halides	T, P, MW, Tb, Pc, Tc, Vc, $\rho_{c}$, Zc, and acentric factor (*ω )*	Decision Tree (DT)	0.9449	0.0150	–	–	[70]
				Multilayer Perceptron (MLP)	0.8720	0.0228	–	–	
				Random Forest (RF)	0.9795	0.0091	–	–	
				Extremely Randomized Trees (ET),	0.9862	0.0075	–	–	
Amar et al. (2023)	1484	*15 alkanes*: Methane, ethane, propane, butane, pentane, hexane, heptane, octane, decane, dodecane, hexadecane, eicosane, octacosane, hexatriacontane, hexatetracontane	T, P, Tc, Pc, and acentric factor (*ω )*	Multilayer Perceptron (ANN)	0.9959	0.004	–	–	[3]
				Cascaded Forward Neural Network (ANN)	0.9969	0.0035	–	–	
				committee machine intelligent system (CMIS)	0.9972	0.0033	–	–	
Hadavimoghaddam et al. (2023)	673	*26 Alcohols*: Methanol, Ethanol, 1-Propanol, 1-Butanol, 1-Pentanol, 1-Hexanol, 1-Heptanol, 1-Octanol, 1-Nonanol, 1-Decanol, 1-Undecanol, 1-Dodecanol, 1-Hexadecanol, 2-Propanol, 2-Butanol, 2-Ethylhexanol, 2-Methyl-1-propanol, Cyclohexanol, 1,2-Ethanediol, 2-Methoxyethanol, 1,3-Propanediol, 2-Butoxyethanol, 2-Ethoxyethanol, 1,4-Butanediol, 2,2’ -Oxybisethanol, 2-(2-Methoxyethoxy)ethanol	T, P, Tc, Pc, and MW	Genetic programming (GP)	0.9691	0.0067	–	–	[73]
				Group method of data handling (GMDH)	0.9841	0.0048	–	–	
Tatar et al. (2022)	1845	*15 alkanes*: Methane, ethane, propane, butane, pentane, hexane, heptane, octane, decane, dodecane, hexadecane, eicosane, octacosane, hexatriacontane, hexatetracontane	T, P, Tc, Pc, Mw, boiling point (Tb), and acentric factor (*ω )*	Decision tree	0.9998^a^	0.0009^a^	0.002^a^	–	[4]
				Random forest	0.9935^a^	0.0051^a^	0.0027^a^	–	
				Gradient boosting	0.9964^a^	0.0038^a^	0.0023^a^	–	
				Extremely randomized trees	0.9944^a^	0.0047^a^	0.0028^a^	–	
Hosseini (2022)	285	*17 Alcohols*: Methanol, Ethanol, 1-Propanol, 1-Butanol, 1-Pentanol, 1-Hexanol, 1-Octanol, 2-Propanol, 2-Butanol, 2-Ethylhexanol, 1,2-Ethanediol, 2-Methoxyethanol, 2-Butoxyethanol, 2-Ethoxyethanol, furfuryl alcohol, allyl alcohol, Isobutanol	P and T	17 different Arrhenius correlations for 17 alcohols (each alcohol has specific correlation)	0.9958	0.0018	–	–	[74]
Zhou et al. (2022)	194	*7 Alcohols*: methanol, ethanol, 1-propanol, 2-propanol, allyl alcohol, 1-butanol, and furfuryl alcohol	T, P, Tc, Pc, and acentric factor (*ω )*	LSSVM	0.9988	–	–	–	[75]
				ANFIS2	0.9988	–	–	–	
				ANFIS3	0.9976	–	–	–	
				MLP	0.9982	–	–	–	
				CFF	0.9982	–	–	–	
				GR	0.9958	–	–	–	
				RBF	0.9441	–	–	–	
Mohammadi et al. (2022)	673	*26 Alcohols*: Methanol, Ethanol, 1-Propanol, 1-Butanol, 1-Pentanol, 1-Hexanol, 1-Heptanol, 1-Octanol, 1-Nonanol, 1-Decanol, 1-Undecanol, 1-Dodecanol, 1-Hexadecanol, 2-Propanol, 2-Butanol, 2-Ethylhexanol, 2-Methyl-1-propanol, Cyclohexanol, 1,2-Ethanediol, 2-Methoxyethanol, 1,3-Propanediol, 2-Butoxyethanol, 2-Ethoxyethanol, 1,4-Butanediol, 2,2^’^ -Oxybisethanol, 2-(2-Methoxyethoxy)ethanol	T, P, Tc, Pc, and MW	deep echo state network (DeepESN)	0.9957	0.0023	–	–	[76]
				extreme gradient boosting (XGBoost)	0.9946	0.0021	–	–	
				extreme learning machine (ELM)	0.9943	0.0022	–	–	
				multivariate adaptive regression splines (MARS)	0.9859	0.0040	–	–	
Hadavimoghaddam et al. (2022)	1332	*32 hydrocarbons*: Ethane, propane, butane, hexane, heptane, octane, decane, dodecane, hexadecane, eicosane, octacosane, hexatriacontane, hexatetracontane, 2,2,4 trimethylpentane, ethene, 1-hexene, 1-heptene, 1-octene, benzene, toluene, ethylbenzene, m-xylene, cumene, 1,2,4 trimethylbenzene, diphenylmethane, cyclohexane, methylcyclohexane, naphthalene, 1,2,3,4 tetrahydronaphthalene, phenanthrene, pyrene, squalane	T, P, Tc, Pc, and Mw	Genetic programming	0.9861	0.0132	–	–	[5]
				Group method of data handling	0.9687	0.0198	–	–	
Mohammadi et al. (2021)	919	*26 hydrocarbons*: butane, hexane, heptane, octane, decane, dodecane, hexadecane, eicosane, octacosane, hexatriacontane, hexatetracontane, 2,2,4 trimethylpentane, 1-octene, benzene, toluene, ethylbenzene, m-xylene, cumene, 1,2,4 trimethylbenzene, cyclohexane, methylcyclohexane, naphthalene, 1,2,3,4 tetrahydronaphthalene, phenanthrene, pyrene, squalane	T, P, Tc, Pc, and Mw	Extreme gradient boosting	0.9998	0.0007	–	1.81	[6]
				Adaptive boosting support vector regression	0.9995	0.0012	–	3.40	
				Gradient boosting with categorical features support	0.9993	0.0015	–	4.7	
				Light gradient boosting machine	0.9940	0.0045	–	3.51	
				Multi-layer perceptron	0.9917	0.0053	-	6.01	
Jiang et al. (2021)	278	*11 aromatic/cyclic hydrocarbons*: cyclohexane, toluene, tetrahydrofuran, 1,4-Dioxane, 1-methyl-2-pyrrolidone, benzene, naphthalene, phenanthrene, pyrene, methylcyclohexane, quinoline,	T, P, Tc, Pc, and acentric factor (*ω )*	Adaptive neuro-fuzzy inference systems	0.9966	0.0052	0.00002	7.88	[77]
				Artificial neural networks	0.9953	0.0062	0.00003	8.77	
				Least-squares support vector machines	0.9950	0.0063	0.00004	13.7	

^a^These values have been reported for training set

In our previous study [72], a novel approach including MLR method was introduced for predicting solubility of H_2_ in 32 hydrocarbons by employing a distinct set of molecular descriptors, diverging from the conventional use of critical temperature (T_c_), critical pressure (P_c_), molecular weight (M_w_), and similar common variables. To the best of our knowledge, this work remains the only study in the literature that has utilized alternative descriptors specifically designed to better reflect structural variations in hydrocarbons. Through a recent literature review (see Table 1), two major limitations were identified in earlier studies. Firstly, the molecular variables employed to model were largely uniform across different works predominantly limited to T_c_, P_c_, and M_w_, failing to sufficiently capture the diversity in chemical structures. In our former study [72], we have showed that such structural variables (i.e., critical properties) are not sufficient for the prediction of solubility of H_2_ in hydrocarbons, particularly in high values of solubility of H_2_. Secondly, despite the excellent statistical metrics reported (e.g., R^2^, RMSE, MSE, and AARD%), simpler ML technique like MLR, were notably underutilized. Most previous studies relied on complex, black-box ML models that, while effective, pose significant interpretability and accessibility challenges for wider adoption. Moreover, an imbalance was often observed between the number of descriptors and the structural diversity of datasets; for instance, studies involving 15 and 11 hydrocarbon structures frequently used three or more descriptors, which may have led to overfitting or misleading performance metrics [3, 4, 77]. In contrast, the current work builds upon the previous studies by extending the dataset to 101 structurally diverse chemicals. This is the largest of its kind to date and emphasizes both methodological simplicity and descriptor-to-structure proportionality, aiming for improved robustness and broader usability. Above missing points were obviously clear in [70]. For example, while the reported statistical parameters for the test set (R^2^ = 0.9343 and RMSE = 0.0136) were reasonably acceptable, they were significantly lower than those for the training set (R^2^ = 1.000 and RMSE = 0.0003). This discrepancy suggests possible overfitting, likely due to the use of complex black-box models for predicting solubility of H_2_. To mitigate such flaws and improve model generalizability, it is more effective to develop a MLR model using an appropriate number of molecular descriptors alongside T and P variables. Because molecular descriptors are straightforward to compute and more reflective of structural characteristics than critical properties (such as T_c_ and P_c_), their use can help achieve more balanced performance between training and test sets. It should also be mentioned that four chemicals such as 1-naphthol, dibenzyltoulene, n-ethylcarbazole, and eugenol had been excluded from the database in the most recent study [70], due to the unavailability of critical properties. Unavailability of critical properties is one of biggest problems that researchers face when they want to screen huge number of chemicals for the specific application. In this study, we aim to address these points.

The Quantitative Structure–Property Relationship (QSPR) approach is widely employed to establish both quantitative and qualitative links between macroscopic properties such as solubility of H_2_ and molecular structure, using calculated molecular descriptors [72, 78–80]. In contrast to previous studies (see Table 1), the QSPR methodology allows for a more comprehensive exploration. It enables the identification of meaningful descriptors from a vast descriptor pool, offering deeper insight into the relationship between chemical structures and solubility of H_2_. Variables selection (or descriptors selection) among of variables pool is one of the major tasks should be taken carefully into account when researchers intend to consider the structural effects in their study. This task is almost ignored by former studies. As a computational approach, the QSPR method offers a powerful means to accurately predict solubility of H_2_ in a wide range of chemicals. This capability is particularly valuable for advancing thermodynamic models such as PC-SAFT and Peng–Robinson, as well as optimizing various chemical processes. In this study, the QSPR approach is implemented using the widely recognized software QSARINS [81–83], which provides robust internal and external validation tools to ensure model reliability and generalizability.

This study aims to provide an accurate, transparent, and generalizable model for predicting solubility of H_2_ in a broad spectrum of chemicals using the QSPR approach. In response to the limitations observed in previous studies including the overuse of critical properties (Tc, Pc, and ω) as descriptors, the underrepresentation of simpler and more interpretable models like MLR, and the mismatch between descriptor quantity and dataset diversity, this work builds upon our previous research [72] by significantly expanding the dataset to 101 structurally diverse chemicals across wide T and P ranges (totally, 4345 datapoints). In contrast to our previous study [72], the present work explicitly accounts for the influence of hydroxyl functional groups, enabling the development of a model capable of accurately predicting the target property for structures containing such functionalities and or oxygen atoms. Notably, the new model also reduces the prediction error for the H_2_-octanedecane system experimentally measured in our group, despite this system not being included in any of the datasets listed in Table 1 for either training or evaluation. Given that our earlier model [72] exhibited a deviation for this system, the improvements achieved here highlight the enhanced accuracy and broader applicability of the newly proposed correlation. The main aim is to develop an MLR-based model that balances accuracy and interpretability, and to benchmark it against more complex ML methods used in the literature. By refining input selection, ensuring descriptor-to-structure proportionality, and validating models rigorously, this work contributes to the advancement of predictive modeling for solubility of H_2_, with direct implications for H_2_ storage, thermodynamic modeling, and industrial process design.

## Method

As in our previous research [72] and as in [70], the solubility of H_2_ (i.e., x) was transformed using the natural logarithm (i.e., ln x) prior to modeling. This transformation was adopted for both theoretical and practical reasons. First, the distribution of x is typically lognormal, with many values approaching zero; converting to ln x helps normalize the distribution and stabilize variance, which enhances model performance and statistical reliability. Second, this transformation helps to maintain the physical realism of the model's predictions. Since solubility of H_2_ is often very small and strictly positive, applying a logarithmic transformation (i.e., ln x) ensures that the linear model does not produce nonphysical negative values, which can otherwise result from extrapolation in regression models like MLR. Thus, transforming x into ln x not only improves the statistical properties of the data but also preserves the physical integrity of the model outputs. Similarly, natural logarithmic transformations of T and P (ln T and ln P) were applied, as previously established in our former study [72], to maintain consistency and improve linearity between input variables and the target output.

### Basic theory

After converting x into ln (x), the effects of T, P, and molecular structures on solubility of H_2_ were included as the independent variables as below (Eq. 1):

1 $$ {\mathrm{ln}}({\mathrm{x}}) = {\mathrm{a}}_{{1}} {\mathrm{ln}}\left( {\mathrm{P}} \right) + {\mathrm{a}}_{{2}} {\mathrm{ln}}\left( {\mathrm{T}} \right) + {\mathrm{F}}\left( {{\mathrm{descriptors}}} \right) + {\mathrm{a}}_{{3}} $$

where ‘a_1_’, ‘a_2_’, and ‘a_3_’ are adjustable parameters. In this study, the most important descriptors (i.e., F(descriptors) in Eq. 1) were used to take into account the effect of chemical structural variations and the effects of operational conditions (i.e., T and P) are considered using ‘ln T’ and ‘ln P’, respectively. In the Supporting Information Excel file (Table Sheet 1), the linear correlation between ln x and ln P is visually illustrated for several solvents as examples.

### Dataset

Based on the compiled experimental data [8–69], the current study presents the largest and most structurally diverse dataset to date, both in terms of chemical variety (i.e., 101 chemicals) and number of datapoints (i.e., 4345), compared to previously reported datasets in Table 1. As can be seen in Table 1, the biggest dataset including 96 chemicals and 3726 datapoints had been used for training and evaluation of models [70]. The detail of this dataset can be found in Supporting Information Excel file (Table Sheet 1). To ease the comparison with former studies and development of the reliable models, data-curation and data mining (like [70]), must be done on this dataset. For example, 59 duplicated datapoints, 3 inconsistent datapoints, 476 datapoints with very high values of P and 8 datapoints with very high values of T were excluded from this dataset. Alongside these excluded datapoints, 30 further datapoints are excluded. The details of all the excluded datapoints can be found in Supporting Information Excel file (Table Sheet 2). In total, a dataset including 3761 datapoints plus our 8 previous experimental x (solubility of H_2_ in octadecane) at wide ranges of T (88–623 K) and P (1–299 bar) was finally used for training and evaluation of models in the present study.

### Former available models

As can be seen in Table 1, there are many studies which have been conducted for the prediction of solubility of H_2_ in different types of chemicals. Among different ML algorithms in Table 1, there are three white-box studies: by our research group [72], Hadavimoghaddam et al. [5], and [73], which have been performed on sufficient variation of chemicals. Also, there is one black-box study by Foroughizadeh et.al [70] with which our white-box study’s results will be compared. The MLR-QSPR model is a white-box approach, and it is rational to make comparison the obtained outputs of present study with the most qualitative and quantitative white-box models with enough variation of chemicals [5, 72, 73]. As already shown in Table 1, these white box models were built using 32, 32, and 26 different structures simultaneously consider the effects of structural variations of chemicals, T, and P. The used molecular descriptors were four PaDEL descriptors (i.e., ATSC0m, MATS1e, minssCH2, ETA_Beta_s) in our former study [72], while T_c_, P_c_, and M_w_ were structural variables in [73] studies. Hadavimoghaddam et al. [5, 73] assessed the prediction capability of their predictive GP-models using common statistical parameters like R^2^ and RMSE.

### Data-driven QSPR approach

#### Model development and calculation of molecular descriptors

As can be seen in Eq. (1), ln (x) is simultaneously a function of chemical descriptors alongside T and P. So, the molecular descriptors numerically expressing the molecular features (characteristics) must be calculated first. In this study, 2D, 1D, and 0D descriptors were attempted first which were independent of the geometry-optimization of molecular structures of chemicals. Before calculation of such descriptors, each molecular structure of chemicals was drawn in ChemBioDraw-Ultra [84]. Then, the drawn structures in ‘MDL mol’ format fed to PaDEL-Descriptor software [85] for the calculation of descriptors. Descriptors with constant or almost constant values for each chemical were eliminated. Finally, a pool of 1239 descriptors remained. For model construction, the suitable descriptors must be selected from the PaDEL descriptors pool. There are well-known methods of descriptors selection such as artificial neural network (ANN) [86], replacement method (RM) [87], genetic algorithm (GA) method [88]. In this study, Genetic Algorithm (GA) was used to build MLR QSPR models-based PaDEL descriptors. The details of the GA-MLR algorithm can be found elsewhere [89, 90]. QSARINS software was applied to develop the GA-MLR models.

#### Evaluation of QSPR-models using statistical parameters

The goodness-of-fit of QSPR model should be carefully checked using the standard statistical parameters, including leave-one-out cross-validated coefficient of determination (Q^2^_LOO_-_CV_), coefficient of determination (R^2^), adjustable coefficient of determination (R^2^_Adj_), root mean square error (RMSE), Fisher function (F), average absolute relative deviation (%AARD), average absolute deviations (AAD), standard residual (S), and maximum (or critical) leverage (h^*^). More detailed information regarding the statistical parameters applied in this study can be found in Table 2 (Eqs. 2–10).

**Table 2:** Used statistical parameters in the present study

Introduced parameters	Introduced parameters equations	Eqs. No
Coefficient of determination	${\mathrm{R}}^{{2}} = {1}{-}\frac{{\mathop \sum \nolimits_{{{\mathrm{i}} = 1}}^{{\mathrm{n}}} \left( {{\text{ Y}}_{{\mathrm{i}}}^{{{\mathrm{exp}}}} - {\mathrm{Y}}_{{\mathrm{i}}}^{{{\mathrm{pre}}}} { }} \right)^{2} }}{{\mathop \sum \nolimits_{{{\mathrm{i}} = 1}}^{{\mathrm{n}}} \left( {{\text{ Y}}_{{\mathrm{i}}}^{{{\mathrm{exp}}}} - {\overline{\mathrm{Y}}}_{{\mathrm{i}}} { }} \right)^{2} }}$	(2)
Adjustable coefficient of determination	${\mathrm{R}}^{{2}}_{{{\mathrm{Adj}}}} = {1}{-}\left( {{1} - {\mathrm{R}}^{{2}} } \right) \times \left( {\frac{{{\mathrm{n}} - 1}}{{{\mathrm{n}} - {\mathrm{p}} - 1}}} \right)$)	(3)
Leave-one-out cross-validated coefficient of determination	${\mathrm{Q}}^{{2}}_{{{\mathrm{LOO}} - {\mathrm{CV}}}} = {1}{-}\frac{{\mathop \sum \nolimits_{{{\mathrm{i}} = 1}}^{{\mathrm{n}}} \left( {{\text{ Y}}_{{\mathrm{i}}}^{{{\mathrm{exp}}}} - {\mathrm{Y}}_{{\mathrm{i}}}^{{{\mathrm{pre}} - {\mathrm{CV}}}} { }} \right)^{2} }}{{\mathop \sum \nolimits_{{{\mathrm{i}} = 1}}^{{\mathrm{n}}} \left( {{\text{ Y}}_{{\mathrm{i}}}^{{{\mathrm{exp}}}} - {\overline{\mathrm{Y}}}_{{\mathrm{i}}} { }} \right)^{2} }}$	(4)
Fisher function	${\mathrm{F}} = \frac{{\mathop \sum \nolimits_{{{\mathrm{i}} = 1}}^{{\mathrm{n}}} \left( {{\text{ Y}}_{{\mathrm{i}}}^{{{\mathrm{pre}}}} - \overline{Y}_{{\mathrm{i}}} } \right)^{2} /{\mathrm{p}}}}{{\mathop \sum \nolimits_{{{\mathrm{i}} = 1}}^{{\mathrm{n}}} \left( {{\text{ Y}}_{{\mathrm{i}}}^{{{\mathrm{exp}}}} - {\mathrm{Y}}_{{\mathrm{i}}}^{{{\mathrm{pre}}}} { }} \right)^{2} /{\mathrm{n}} - {\mathrm{p}} - 1}}$	(5)
Standard residual	${\mathrm{S}} = \sqrt {\frac{{\mathop \sum \nolimits_{{{\mathrm{i}} = 1}}^{{\mathrm{n}}} \left( {{\text{ Y}}_{{\mathrm{i}}}^{{{\mathrm{exp}}}} - {\mathrm{Y}}_{{\mathrm{i}}}^{{{\mathrm{pre}}}} { }} \right)^{2} }}{{{\mathrm{n}} - {\mathrm{p}} - 1}}}$	(6)
root mean square error (RMSE)	${\mathrm{RMSE}} = \sqrt {\frac{{\mathop \sum \nolimits_{{{\mathrm{i}} = 1}}^{{\mathrm{n}}} \left( {{\text{ Y}}_{{\mathrm{i}}}^{{{\mathrm{exp}}}} - {\mathrm{Y}}_{{\mathrm{i}}}^{{{\mathrm{pre}}}} { }} \right)^{2} }}{{\mathrm{n}}}}$	(7)
Average absolute deviation	${\mathrm{AAD}} = \frac{{\mathop \sum \nolimits_{{{\mathrm{i}} = 1}}^{{\mathrm{n}}} \left( {\left| {{\mathrm{Y}}_{{\mathrm{i}}}^{{{\mathrm{exp}}}} - {\mathrm{Y}}_{{\mathrm{i}}}^{{{\mathrm{pre}}}} } \right|} \right) }}{{\mathrm{n}}}$	(8)
Average absolute relative deviation %	${\mathrm{AARD}}\% = \frac{{\mathop \sum \nolimits_{{{\mathrm{i}} = 1}}^{{\mathrm{n}}} \left( {|{\mathrm{Y}}_{{\mathrm{i}}}^{{{\mathrm{exp}}}} - {\mathrm{Y}}_{{\mathrm{i}}}^{{{\mathrm{pre}}}} |} \right)/{\mathrm{Y}}_{{\mathrm{i}}}^{{{\mathrm{exp}}}} }}{{\mathrm{n}}} \times 100$	(9)
Maximum leverage	${\mathrm{h}}^{*} = {3}\left( {{\mathrm{p}} + {1}} \right)/{\mathrm{n}}$	(10)

Y_i_^exp^, Y_i_^pre^, ${\overline{\mathbf{Y}}}_{{\mathbf{i}}}$, n, and p demonstrate experimental values, predicted values, average experimental values, the number of the experimental dataset, and the number of employed descriptors, respectively

Applicability of Domain (AD) is a vital concept of QSPR approach, but often ignored in previous studies. According to [91] it allows: (1) the uncertainty in prediction (2) the extent of extrapolation of QSPR models [92, 93]. In order to predict solubility of H_2_ in new structures of chemicals, it is essential that new structures lie within the same AD space. This means that the new chemicals are physicochemically, biologically, or structurally similar with molecules used for model development (i.e., training set). The more space of AD, the more reliable predictions of new chemicals. To carry out the external validation using validation set, it is essential to ensure that the validations set of molecules is inside of QSPR model’s AD [94].

The space of AD can be specified using two main parameters: (1) the leverage values (h_i_) (2) the standardized residual (SDR) and. SDR is defined in Eq. (11):

11 $$ {\mathrm{SDR}} = \frac{{{\mathrm{Y}}_{{\mathrm{i}}}^{{{\mathrm{exp}}}} - {\text{ Y}}_{{\mathrm{i}}}^{{{\mathrm{pre}}}} }}{{\sqrt {\frac{{\mathop \sum \nolimits_{m = 1}^{n} ({\mathrm{Y}}_{{\mathrm{i}}}^{{{\mathrm{exp}}}} - {\text{ Y}}_{{\mathrm{i}}}^{{{\mathrm{pre}}}} )^{2} }}{n}} }} $$

where h_i_ represents a measure of a molecule’s distance from the center of the training set. It is needed to determine whether new chemicals are within the applicability of domain of the developed QSPR model or not. The parameter can be calculated with Eq. (12).

12 $$ {\mathrm{h}}_{{\mathrm{i}}} \left( {{\text{or Leverage }}\left( {\mathrm{i}} \right)} \right) = {\mathrm{z}}_{{\mathrm{i}}} \cdot \left( {{\mathrm{Z}}_{{\mathrm{i}}}^{{\mathrm{T}}} {\mathrm{Z}}_{{\mathrm{i}}} } \right)^{{ - {1}}} \cdot {\mathrm{z}}_{{\mathrm{i}}}^{{\mathrm{T}}} $$

where z_i_, Z are the descriptor row vector of point i and a n *×* p matrix of descriptors for chemicals derived from the training set, respectively. AD of developed QSPR models can be obtained in QSARINS software for each model and maximum leverage (i.e., h^*^) can be calculated using Eq. (10).

#### External and internal validations

To select and develop the QSPR model, it is necessary to perform internal and external validations on the training (approx. 75% of main dataset) and test (approx. 25% of main dataset) sets, respectively. Regarding the internal validation, leave one out-cross validation (LOO-CV), Y-scrambling, leave multi out-cross validation (LMO-CV) methods should be carried out on the developed QSPR model. These methods were performed on the training set only. Regarding the external validation, the prediction capability of developed QSPR model was assessed using a test set. Both of internal and external validations of QSPR models can be carried out in QSARINS software one by one, due their high importance. To ensure consistency and avoid potential bias, the same chemical structures previously assigned to the test set by [70] were selected as the test set in this study. This approach facilitates more reliable and direct comparisons between the models.

## Results and discussion

Prior to presenting the main results, it is crucial to determine the optimal number of variables including T, P, and molecular descriptors to be used in developing the MLR models. This can be effectively evaluated through a breaking plot analysis. Breaking plot has been shown in Supporting Information Excel file (Sheet 3). Based on the breaking plot analysis, it was observed that a model incorporating six variables, namely ln T, ln P, and four critical property descriptors, exhibited comparable predictive accuracy to models developed with a larger number of variables. For example, a model including six optimally selected variables (R^2^ = 0.940) has almost the same R^2^ value as a model including nine variables (R^2^ = 0.945). This means that adding new variables alongside the six existing ones not only fails to improve the model's accuracy but is also meaningless. Nevertheless, 10 variables (T and P, and 8 properties as input set 1) were surprisingly applied in the development of nonlinear models in most recent study by Foroughizadeh et.al [70], for a dataset including 96 chemicals and 3726 datapoints. Moreover, more than 10 variables have been irrationally used in other models as input sets 2 and 3 [70]. Therefore, it is strongly recommended that for a dataset with 96 structural variations, 6 critical properties alongside 2 operational variables (T and P) must be applied, in maximum, and using properties higher than this one is irrational. On the contrary, when PaDEL descriptors are used instead of critical properties, a slight improvement on R^2^ was observed though (see Supporting Information Excel file (Sheet 3)). Another important consideration is the use of a non-flexible input variable pool, restricted to only 10 predefined variables. This limitation constrains the model development process and may hinder the identification of a highly accurate predictive model. In QSPR studies, variable selection is a critical step, and a limited descriptor space significantly reduces the ability to explore and identify the most informative variables. And even, there is an intercorrelation between some of variables. For example, there is a very high correlation (R^2^ = 0.99) between M_W_ and V_C_ for 96 of studied chemicals which may lead to overfitting, if they use in a model, simultaneously.

**Fig. 1:**
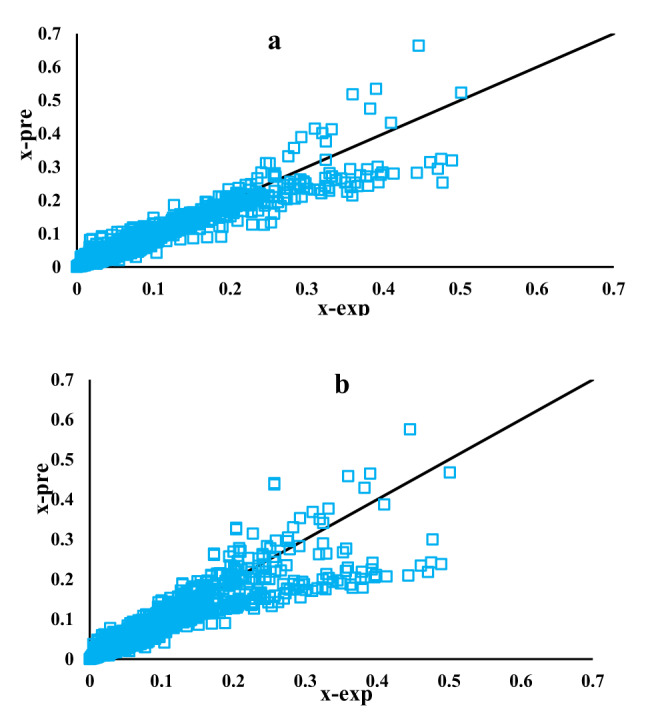
Predicted versus experimental values using models with 9 variables including **a** PaDEL descriptors and **b** critical properties

To demonstrate the limitations of critical properties compared to PaDEL descriptors, it is essential to evaluate and compare the predictive performance of models developed using two distinct types of variables: (1) critical properties and (2) PaDEL descriptors, applied to the same dataset. To create the same dataset, 73 datapoints of 4 excluded chemicals by [70], were also discarded from our final dataset including 3761 datapoints. Critical property data were missing for four chemicals, leaving no option but to remove them in order to construct the same dataset. Therefore, the same dataset comprising 96 chemicals and 3,688 data points was used for the comparison. The full dataset is detailed in the Supporting Information Excel file (Table Sheet 4). As can be seen in Fig. 1, the MLR model including the PaDEL descriptors, showed more prediction capability (R^2^ = 0.93, RMSE = 0.0172) than model including critical properties (R^2^ = 0.85, RMSE = 0.0249), in overall status. In accordance with our former results [72], it has been again shown that when a predictive MLR-model has the critical properties alongside ln T and ln P variables, weak performance must be expected for high values of solubility of H_2_ in chemicals. While an MLR-model including PaDEL descriptors showed equal performance for both low and high values of solubility of H_2_ in chemicals. Therefore, using of such descriptors which express the chemicals’ features, is better to use in the MLR or even nonlinear models which had been done by [70].

### Developed MLR-QSPR models

In the present study, the dataset including 100 distinct structures of 11 types of chemicals and 3761 datapoints was categorized into the training and test sets, with 75/25 ratio, for performing of internal and external validations, respectively. All datapoints of 24 chemicals including 20 chemicals which had been already set aside into test set by [70] and four chemicals which had been already excluded by [70]), were set deliberately aside into test set. The detail of training set including 2570 datapoints and test set including 1191 datapoints can be found in Supporting Information Excel file (Table Sheet 5). The primary objective of this classification is not only to evaluate the predictive performance of the MLR-QSPR model on new chemicals, but also to enable a comprehensive comparison with the most recent and extensive dataset. Also, another important objective is to check the model’s performance for the prediction of solubility of H_2_ in four excluded chemicals by [70]. An MLR-QSPR model with maximum allowed variables (i.e., 6 PaDEL descriptors plus ln T and ln P) using above training set is suggested here. The suggested MLR-QSPR model which was built in training and test status (i.e., Eq. 13) is indicated in Table 3.

**Table 3:** The suggested MLR-QSPR models for the training set including 2570 datapoints

Kind of structural variable	Number of datapoints and chemicals in training set	Model^a^	Eq. No
PaDEL	2570 and 76	ln(x) = 1.8101 ln(T) + 1.0011 ln(P) + 0.0285 nBondsS3 + 0.6652 Mi − 0.0624 SsOH + 1.1123 minHBd − 7.9672 ETA_dEpsilon_D – 4.8234 ETA_BetaP_s − 20.9520	(13)

^a^The units of T and P are K and bar, respectively

The values of statistical parameters of Eq. (13) are reported in Table 4.

**Table 4:** The values of statistical parameters of suggested MLR-QSPR models (ln-based) for both of training and test sets

Eqs. No	Sets	Number of datapoints and chemicals	R^2^	R2-Adj	Q2-LOO	Q2-LMO	F	S	RMSE
(13)	Training	2570 and 76	0.9616	0.9614	0.9612	0.9613	8009	0.2620	0.2615
	Test	1191^a^ and 24	0.9282	–	–		–	–	0.3096

^a^This one also includes 73 datapoints of 4 chemicals that their critical properties were not available

**Fig. 2:**
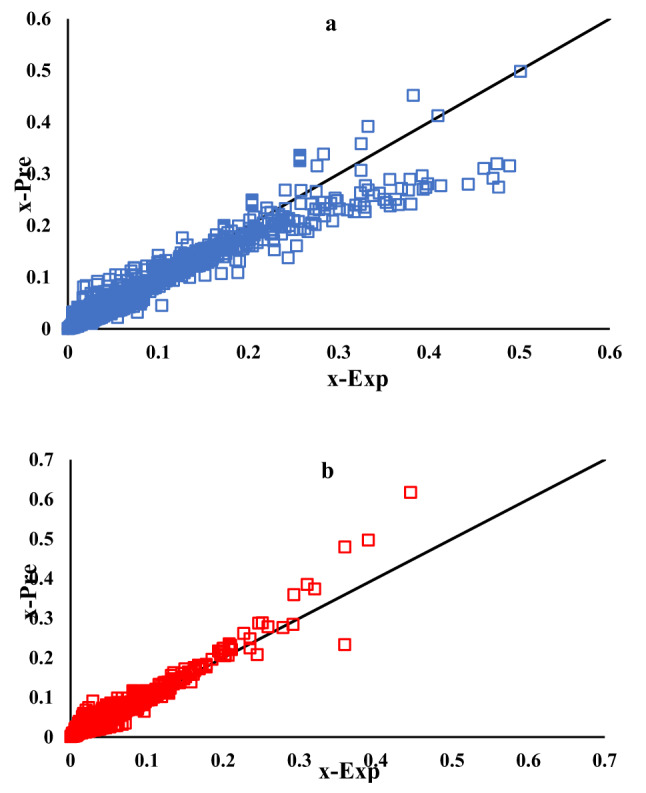
Predicted versus experimental values for both of **a** training and **b** test sets using PaDEL descriptors (Eq. 13)

As exposed in Table 4, the value of Q^2^_LOO_ (as internal validation) of MLR-QSPR model with PaDEL descriptors was high enough which is confirming that the model has acceptable capability for prediction of solubility of H_2_ in studied chemicals of our dataset at the wide ranges of T and P. Also, Y-scrambling and LMO-CV techniques have been conducted to the training set in the QSARINS software for this MLR-QSPR model and results verified (or proved) the validity of the model. As external validation, it is also demonstrated that solubility of H_2_ in those chemicals of test set predicted with high accuracy based on the obtained values of RMSE. The proposed MLR-QSPR model (i.e., Eq. 13) could take into account the effects of T and P on the x in majority of studied chemicals. The Williams plot for the training and test sets which was obtained using MLR-QSPR model (i.e., Eq. 13), is shown in Supporting Information Excel file (Table Sheet 6). The plot confirms that there were not many outliers in the studied dataset as only three datapoints at given T and P has a leverage value higher than the critical leverage (i.e., h* = 0.011) and their SRD a little bit higher than ± 3. These three datapoints are related to the only present S-containing chemical in the dataset (i.e., carbon disulphide) which is naturally different from others. Although the leverage values of some hydrocarbons were quite higher than the critical leverage (h*), the MLR-QSPR model could predict the x in those chemicals well. The plots of predicted versus experimental values for both of training and test sets which were obtained using MLR-QSPR model (i.e., Eq. 13), are shown in Fig. 2.

To further show the necessity of effective descriptors in the models, two separate MLR-models (1) without any descriptors (i.e., Eq. 14) and (2) with P_c_, T_c_, V_c_, Zc, T_b_, $\rho_{C}$, and M_w_ descriptors (i.e., Eq. 15) have been regressed on our dataset. These two models have been shown in Table 5.

**Table 5:** The built MLR models without and with former descriptors (i.e., with Pc, Tc, Vc, Zc, T_b_, $\rho_{C}$, and Mw) for the training and test status

Without/withdescriptors	Number ofdatapoints andchemicals intraining set	Model	Eq. No
Without	2570 and 76	ln(x) = 0.1595 ln(T) + 1.0934 ln(P) − 8.7623	(14)
With former descriptors	2570 and 76	ln(x) = 1.6482 ln(T) + 0.9858 ln(P) + 0.0230 Mw − 0.0066 Tb − 0.0010 Tc − 0.0190 Pc − 0.0042 Vc − 9.2239 ρ_C − 6.7746 Zc − 9.7478	(15)

The values of statistical parameters of each model (ln-based) are shown in Table 6.

**Table 6:** The values of statistical parameters of MLR-models (ln-based) without and with former descriptors for both of training and test sets

Eqs. No	Sets	Number of datapoints and chemicals	R^2^	R2-Adj	Q2-LOO	F	S	RMSE
(14)	Training	2570 and 76	0.6462	0.6460	0.6455	2344	0.7939	0.7934
	Test	1191 and 24	0.6055	–	–	–	–	0.7329
(15)	Training	2570 and 76	0.9453	0.9451	0.9445	4911	0.3127	0.3121
	Test	*1118*^a^ and 20	0.9137	–	–	–	–	0.3162

^a^This one does not include 73 datapoints of 4 chemicals that their critical properties were not available

**Fig. 3:**
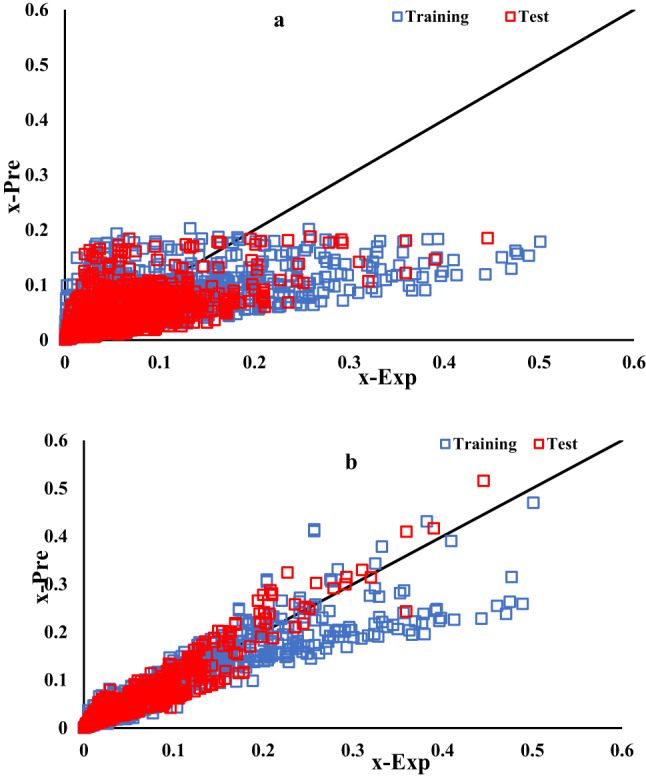
Predicted versus experimental values for both of training and test sets using **a** without any descriptors (Eq. 14) and **b** with Pc, Tc, Vc, Zc, Tb, $\rho_{C}$, and Mw descriptors (Eq. 15)

The plots of predicted versus experimental values for both training and test sets obtained using MLR-models (i.e., Eqs. 14 and 15), are shown in Fig. 3.

A general none-ln based comparison between all the developed models (i.e., Eqs. 13–15) in this study has been performed using some statistical parameters reported in Table 7. The detail can be found in Supporting Information Excel file (Table Sheet 7).

**Table 7:** Statistical parameters of all developed models in this study for both of training and test sets (*none ln -based*)

Eqs. No	Sets	R^2^	RMSE	AAD	AARD%
(13)	Training	0.94	0.0182	0.0083	19.36
	Test	0.95	0.0131	0.0075	22.69
(14)	Training	0.48	0.0519	0.0311	97.33
	Test	0.39	0.0439	0.0302	65.59
(15)	Training	0.86	0.0253	0.0114	23.75
	Test	0.89	0.0188	0.0125	25.68

**Fig. 4:**
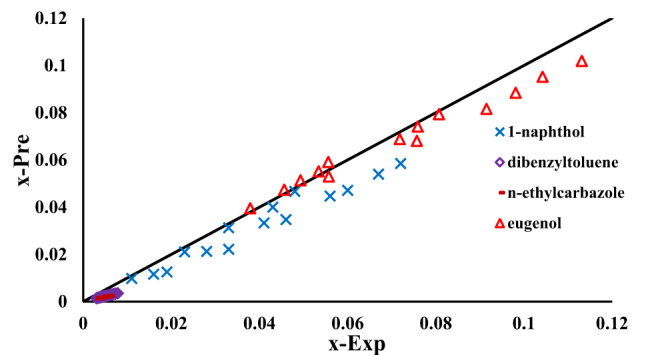
Predicted values (using Eq. 13) of solubility of H_2_ in four chemicals that their critical properties were not available

As can be seen in Table 7 and Fig. 3, both developed MLR-models (i.e., Eqs. 14 and 15) had not acceptable accuracy for the prediction of solubility of H_2_ in chemicals. This point verifies that suggested MLR-QSPR model (i.e., Eq. 13) in the present study, could efficiently predict the target, due to their relevant molecular structure dependent descriptors. While, the prediction capability of Eq. (13) (with 8 variables) has been evaluated for 24 different structures of chemicals as external validation, the obtained RMSE is much lower than reported values for Eq. (15) (with 9 variables) which has been evaluated for 20 structures. This point expresses two major advantages of the present study. The first one is that when a proper set of molecular descriptors is used in an MLR-model for the prediction of solubility of H_2_ in chemicals, reaching to the high accuracy of prediction is easily possible. And this accuracy is also competitive with other accuracies which were obtained using former complicated algorithm of ML (See reported statistical parameters in Table 1). And the second one is that the calculation of this set of descriptors is not limited to some structures of chemicals. It means that these descriptors are simply calculable for any desired structures of chemicals, particularly for those four excluded chemicals by [70]. The good agreement between the predicted x and experimental x in those four chemicals has been exposed in Fig. 4. As already mentioned, the prediction of solubility of H_2_ in those four chemicals was not possible either using proposed MLR model including critical properties in the present study (i.e., Eq. 15) or proposed none-linear models by [70]. Because our previous QSPR model [72] was trained using only hydrocarbon systems, it did not perform effectively in predicting H₂ behavior in those compounds including hydroxyl groups like 1-naphtol and or oxygen atoms like eugenol. Importantly, the final model identifies the descriptor SsOH, the sum of atom-type E-State values associated with hydroxyl groups, as a key parameter governing the influence of –OH functionalities. This demonstrates that the new framework can explicitly capture structural effects that were entirely absent from our previous study [72], which did not include any descriptors capable of representing hydroxyl groups. The emergence and significance of SsOH therefore highlight a major advancement of the present model in recognizing and quantifying functional-group contributions.

**Fig. 5:**
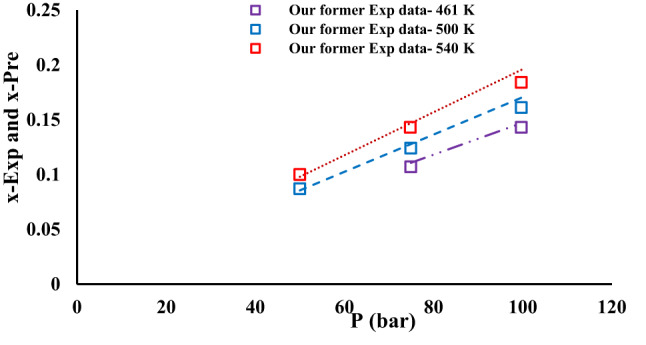
Predicted values by our MLR-QSPR model (Eq. 13) and our former experimental values [8] at three T and different P

For further evaluation, we tried to investigate the prediction capability of our MLR-model (i.e., Eq. 13) for a system that was not earlier considered (see Table 1). As can be seen in Fig. 5, the predicted values by our MLR-QSPR model had an excellent agreement with our former experimental data [8]. The predictive accuracy of our new model (Eq. 13) is notably superior to that of our earlier model [72], achieving more than a twofold decrease in AARD% for H_2_-octanedecane system. The detail can be found in Supporting Information Excel file (Table Sheet 8).

To introduce the appeared descriptors in the suggested MLR-QSPR model with PaDEL descriptors (i.e., Eq. 13), the definition of each above descriptor has been listed in Table 8.

**Table 8:** Definition of each PaDEL-descriptor and critical property

Model	Descriptors	Definition	Ref
Equation (13)	nBondsS3	Total number of single bonds (excluding bonds to hydrogens and aromatic bonds)	[85]
	Mi	Mean first ionization potentials (scaled on carbon atom)	[85]
	SsOH	Sum of atom-type E-State: -OH	[85]
	minHBd	Minimum E-States for (strong) Hydrogen Bond donors	
	ETA_dEpsilon_D	A measure of contribution of hydrogen bond donor atoms	[85]
	ETA_BetaP_s	A measure of electronegative atom count of the molecule relative to molecular size	[85]
Equation (15)	MW(g/mol)	Molecular weight	[70]
	Tc (K)	Critical temperature of solvent	[70]
	Pc (MPa)	Critical pressure of solvent	[70]
	Zc	critical compressibility factor	[70]
	Vc	critical volume	[70]
	Tb	normal boiling point	[70]
	ρc	critical density	[70]

### A comparison with former available models

The most recent study which has been done on the structurally divers dataset (i.e., 96 chemicals and 3726 datapoints, is [70]. For this regard, the results of white-box model of the present study should be fairly (unbiasedly) compared with proposed results in that study which applied black-box methods. To compare the prediction capability of models, RMSE parameter has been reported in Table 9 for each specified chemical which had been set aside into the test set. As can be seen in Table 9 and among of 20 compared chemicals, the obtained values of RMSE using the MLR-QSPR model of the present study for some chemicals were lower than other four black-box models which included many variables. The detail can be found in the Supporting Information Excel file (Table Sheet 9).

**Table 9:** A comparison of obtained values of RMSE using the MLR-model of present study and other black-box models

Chemicals	RMSE-Present Study	RMSE-DT	RMSE-MLP	RMSE-RF	RMSE-ET
1,3-propanediol	***0.001***	0.004	0.001	0.001	0.001
1-methylinaphthalene	0.008	0.021	0.003	0.005	0.003
2-propanol	***0.006***	0.016	0.012	0.010	0.010
Acetonitrile	0.031	0.005	0.002	0.002	0.002
Allyl alcohol	0.025	0.018	0.014	0.009	0.008
Cyclohexane	0.006	0.015	0.007	0.005	0.004
Cyclohexanone	0.006	0.002	0.003	0.002	0.001
Cyclohexene	0.003	0.009	0.002	0.002	0.001
Dimethyl ether	0.017	0.012	0.015	0.012	0.012
Ethane	***0.018***	0.023	0.017	0.019	0.019
Furfural	0.014	0.011	0.003	0.006	0.005
*m*-xylene	***0.006***	0.016	0.008	0.006	0.006
Methyl 2-methylpropanoate	0.003	0.003	0.003	0.002	0.001
Methyl formate	***0.002***	0.004	0.002	0.002	0.002
*n*-hexadecane	0.020	0.034	0.030	0.021	0.017
*n*-hexatriacontane	***0.009***	0.026	0.011	0.018	0.017
*n*-octacosane	***0.009***	0.023	0.016	0.028	0.011
*n*-pentane	0.007	0.017	0.015	0.005	0.004
Propylene	***0.006***	0.011	0.020	0.007	0.007
Tetrahydrofuran	0.002	0.010	0.003	0.002	0.001

Also, the reported average R^2^ value (R^2^ = 0.95) across all 24 chemicals and 1191 datapoints by the MLR-model in the present study (see Table 7) reflects the advantage of model’s prediction capability in comparison of other four used ML algorithms in [70]. However, the prediction capability of these four ML models was only assessed across 20 chemicals and 1125 datapoints. Of course, this point can be attributed to referred unexpected difference between the reported values of R^2^ for the training and test sets, which was discussed already in the introduction section. Although the applying former sophisticated algorithms in [70] using irrelevant 10 variables input, difference between R^2^ values for training and test sets for ET algorithm reached to 0.07 which was quite unexpected. This unexpected point emphasizes that such algorithms are powerful for training the model and for this reason, the R^2^ value for the training set was 1. But the difference between R^2^ values for training and test sets of the present study not only is close to each other, but the R^2^ value of test set is also higher than R^2^ values of training set.

Our former study [72] verified that proposed GP-model by Hadavimoghaddam et al. [5] used some irrelevant descriptors (T_c_, P_c_, and M_w_) to distinguish the effect of hydrocarbons on the solubility of H_2_. Because, when GP-model is examined on a more diverse dataset including hydrocarbons, GP-model may predict negative x in methane. Moreover, GP-model had not good efficiency for the prediction of solubility of H_2_ in light hydrocarbons and it overestimates the prediction solubility of H_2_ in majority of hydrocarbons, due to some irrelevant descriptors (T_c_, P_c_, and M_w_). In our former study [72], we showed the necessity of using a new reliable predictive model including relevant descriptors for prediction of solubility of H_2_ in hydrocarbons, instead of those irrelevant descriptors. Despite the noted limitations of the GP model in predicting H_2_-hydrocarbon systems, we remain motivated to assess its performance for predicting solubility of H_2_ in alcohol-based chemicals, as proposed in [73]. In general comparison, it was tried to predict solubility of H_2_ in all alcoholic chemicals using GP-model which are available in the final dataset (i.e., 3761 datapoints). In another words, the proposed GP-model was applied for prediction of solubility of H_2_ in all alcoholic chemicals (either in training or test set) including 638 datapoints. These predicted values have been compared with predicted values by MLR-model (i.e., Eq. 13). It was found that predicted values using GP-model had higher deviation in comparison with our model for those experimental values which were present in our dataset. The results have been shown in Supporting Information Excel file (Table Sheet 10).

For additional comparison, we aimed to highlight the advantages of the current study relative to our previous work [72]. As illustrated in Fig. 5, the newly developed model trained on a more diverse set of chemical structures demonstrated highly accurate predictions for the H_2_-octadecane system. In contrast, our earlier study [72], which utilized a dataset of only 32 structures, showed comparatively lower prediction accuracy. Regarding the disadvantage of Hosseini’s study [74], it should be mentioned that several parameters in the general form of the model were specifically tuned for each individual alcohol and at distinct T and P conditions. As a result, the reported average R^2^ value across all 17 alcohols reflects these tailored adjustments. While this tuning approach may yield high apparent accuracy, it significantly limits the model’s generalizability. When applied to new alcohols or to conditions outside the originally studied T and P ranges, the model is unlikely to maintain accuracy without re-tuning, thereby restricting its practical applicability for broader predictive use.

As with all data-driven approaches, the reliability and robustness of the developed MLR-QSPR models are inherently dependent on both the quality and quantity of the input data. Future improvements in model performance could be achieved through the inclusion of experimentally measured x for chemicals that are currently underrepresented or absent in the dataset. As an additional step of internal and external validation, several key statistical parameters recommended in the QSTR model by [95], including the concordance correlation coefficient (CCC), Q^2^F1, Q^2^F2, Q^2^F3, the novel metrics (r^2^m-average and r^2^m-delta), as well as R^2^Y-scrambling, Q^2^Y-scrambling, and R^2^Y-randomization provided in Table 10. These parameters confirm the robustness and generalizability of the QSPR model, a level of validation that was omitted in all previous studies listed in Table 1. Moreover, incorporating these quantitative parameters fundamentally distinguishes the present study from previous models (see Table 1) from a predictive standpoint, offering researchers a more rigorous and transparent framework for assessing model performance.

**Table 10:** values of concordance correlation coefficient (CCC), Q2F1, Q2F2, Q2F3, and novel metrics (r2m-avaerage and r2m-delta) as well as R2Y-scrambling, Q2-Y-scrambling, and R2Y-randomization

Eqs. No	Sets	Number of datapoints	CCC	Q2F1	Q2F2	Q2F3	R2m-Aver	R2m-delta	R2-Yscr	Q2-Yscr	R2-Yran
(13)	Training	2570	0.980	–	–	–	–	–	0.003	-0.003	0.003
	Test	1191	0.963	0.928	0.924	0.946	0.896	0.042	–	–	–

## Conclusion

In this study, the largest dataset including 3761 datapoints was used to show the strength of the simplest ML algorithm like MLR for the prediction of solubility of H_2_ in different chemicals. Unlike former molecular variables (i.e., T_c_, P_c_, and M_w_ descriptors), PaDEL descriptors were applied to distinguish the structural effects of chemicals on the solubility of H_2_. The suggested MLR-QSPR model could successfully predict the solubility of H_2_ in chemicals as functions of T, P, and suitable molecular descriptors. The obtained values of statistical parameters (AAD, RMSE, Q^2^_LOO-CV_, and R^2^) of suggested MLR-QSPR model were satisfactory for both training and test sets. The obtained values of AARD% parameter were 19 and 22 for training and test sets, respectively. The external and internal validations verified that the prediction of solubility of H_2_ in different chemicals can be practical with respect to the leverage value of chemicals and AD of MLR-QSPR model. The advantage of MLR-QSPR model in comparisons with other available models in the literature has been shown.

## Supporting Information

This study has only one Supporting Information Excel file. The supporting information excel file of this study includes: SMILEs sheet, details of dataset (Sheet 1), Excluded datapoints from dataset (Sheet 2), Breaking Plot (Sheet 3), the studied datapoints (Sheet 4), the details of training and test sets and values of descriptors (Sheet 5), Williams Plot (Sheet 6), Predicted versus Experimental data (Sheet 7), H_2_-Octadecane system (Sheet 8), calculated RMSE for test set (Sheet 9), GP-model for alcohols (Sheet 10).

## Supplementary Information

This study has only one Supporting Information Excel file. The supporting information excel file of this study includes: SMILEs sheet, details of dataset (Sheet 1), Excluded datapoints from dataset (Sheet 2), Breaking Plot (Sheet 3), the studied datapoints (Sheet 4), the details of training and test sets and values of descriptors (Sheet 5), Williams Plot (Sheet 6), Predicted versus Experimental data (Sheet 7), H_2_-Octadecane system (Sheet 8), calculated RMSE for test set (Sheet 9), GP-model for alcohols (Sheet 10).Below is the link to the electronic supplementary material.

Supplementary Material 1

## Data Availability

No datasets were generated or analysed during the current study.
